# Clinical Significance of Colonoscopy in Patients with Upper Gastrointestinal Polyps and Neoplasms: A Meta-Analysis

**DOI:** 10.1371/journal.pone.0091810

**Published:** 2014-03-17

**Authors:** Zhen-Jie Wu, Yuan Lin, Jun Xiao, Liu-Cheng Wu, Jun-Gang Liu

**Affiliations:** Department of Gastrointestinal Surgery, Affiliated Tumor Hospital of Guangxi Medical University, Nanning, P.R. China; University Hospital Llandough, United Kingdom

## Abstract

**Background:**

Some authors have studied the relationship between the presence of polyps, adenomas and cancers of upper gastrointestinal tract (stomach and duodenum) and risk of colorectal polyps and neoplasms; however, the results are controversial, which may be due to study sample size, populations, design, clinical features, and so on. No meta-analysis, which can be generalized to a larger population and could provide a quantitative pooled risk estimate of the relationship, of this issue existed so far.

**Methods:**

We performed a meta-analysis to evaluate risk of colorectal polyps or neoplasms in patients with polyps, adenomas or cancers in upper gastrointestinal tract comparing with controls. A search was conducted through PubMed, EMBASE, reference lists of potentially relevant papers, and practice guidelines up to 27 November 2013 without languages restriction. Odd ratios (ORs) were pooled using random-effects models.

**Results:**

The search yielded 3 prospective and 21 retrospective case-control studies (n = 37152 participants). The principal findings included: (1) OR for colorectal polyps was 1.15 (95% CI, 1.04–1.26) in the gastric polyps group comparing with control groups; (2) Patients with gastric polyps and neoplasms have higher risk (OR, 1.31 [95% CI, 1.06–1.62], and 1.72 [95% CI, 1.42–2.09], respectively) of colorectal neoplasms comparing with their controls; and (3) Positive association was found between the presence of colorectal neoplasms and sporadic duodenal neoplasms (OR, 2.59; 95% CI, 1.64–4.11).

**Conclusions:**

Findings from present meta-analysis of 24 case-control studies suggest that the prevalence of colorectal polyps was higher in patients with gastric polyps than in those without gastric polyps, and the risk of colorectal neoplasms increases significantly in patients with gastric polyps, neoplasms, and duodenal neoplasms. Therefore, screening colonoscopy should be considered for patients with upper gastrointestinal polyps and neoplasms.

## Introduction

Patients with polyps, adenomas and cancers in upper gastrointestinal (GI) tract (stomach and duodenum) may have synchronous or metachronous polyps and neoplasms in their lower GI tract, especially in the colon and rectum. The mechanisms underlying synchronous or metachronous GI neoplasms remain controversial. One hypothesis is genetic factors. Changing of genes, such as APC, p53, K-ras, hMSH1, and hMSH2, plays important roles in the incidence of gastric and colorectal neoplasms.[1]–[3] Another hypothesis is connected with environmental factors. Many factors, such as H. pylori infection, hyperglycemia, and smoking, influence incidence of both stomach cancer and colorectal cancer.[4]–[6] Duodenal and colorectal adenomas share a common biological behavior that high level of malignant transformation and recurrence after local resection.[7].

When patients undergo a screening gastroduodenoscopy and found polyps, adenomas or cancers in their upper GI tract, clinicians may encounter a problem that whether they should advised those patients to have a colonoscopy screening, the preferred modality for colorectal neoplasms screening,[8] in the near future. We reviewed the recommendations of organizations (including American Cancer Society,[9] American Society for Gastrointestinal Endoscopy,[8] U.S. Multisociety Task Force on Colorectal Cancer,[10] American College of Gastroenterology,[11] British Society of Gastroenterology,[12] World Gastroenterology Organisation[13], and Institute for Clinical Systems Improvement[14]) that produce guidelines for this question. However, we found that no standardized strategies exist on the current recommendations for colorectal polyps and tumors screening in patients with gastric polyps or neoplasms lesions.

Some authors[15]–[38] have studied the relationship between the presence of polyps, adenomas and cancers of upper GI tract and risk of colorectal polyps and neoplasms; however, the results are controversial, which may be due to study sample size, populations, design, clinical features, and so on. No meta-analysis of this issue existed so far. We therefore performed a meta-analysis, which can be generalized to a larger population and could provide a quantitative pooled risk estimate, to evaluate risk of colorectal polyps and neoplasms in patients with polyps, adenomas or cancers in their upper gastrointestinal tract comparing with controls.

## Methods

### Literature Search

We conducted this meta-analysis according to the PRISMA guidelines.[39] The electronic databases PubMed and EMBASE (up to 27 November 2013) were searched for relevant papers using the terms: (duodenum OR duodenal OR gastric OR stomach) AND (colon OR rectum OR rectal OR colorectal) AND (control OR cohort OR retrospective OR prospective OR prevalence). What's more, a manual search of the reference lists of potentially relevant papers and practice guidelines were performed manually to identify any additional studies. Papers published in any language were considered.

### Study Selection

Two authors (Z.W. and Y.L.) independently assessed literature eligibility; discrepancies were discussed and resolved by consensus. The following criteria was used to select fully published studies: (1) studies that examined the prevalence of colorectal polyps or neoplasms in patients with polyps or tumors in their upper GI tract comparing with controls, (2) study cases were patients with polyps or neoplasms in their upper GI tract and controls without the above diseases, (3) studies that have an internal comparison in the same individuals, (4) studies that provided an odds ratio (OR) and the corresponding 95% confidence interval (CI), or provided raw data to calculate these, (5) studies that were case-control or cohort design, and (6) data not duplicated in another manuscript.

### Data Extraction and Quality Assessment

We extracted the following data from included studies: study characteristics (first author name, publication year, country, study period, and study type), cases' characteristics (number of cases and percentage of men, cases' type, and mean age), controls' characteristics (number of controls and percentage of men, types of controls, mean age, case-control matching), main outcome (types of colorectal diseases), and adjustment. Adjusted ORs were selected prior to non-adjusted ORs. For studies that did not report ORs, unadjusted OR and 95%CI were calculated. For studies that reported multiple ORs, such as ORs for both adenomas and cancers, we extracted them as separate OR. If OR for neoplasm was available, we preferred this one.

We assessed the included studies' quality according to the Newcastle-Ottawa quality assessment scale, which evaluated studies' quality in meta-analyses based on three items: patient selection, comparability of controls, and ascertainment of outcome. This quality assessment scale ranges between zero up to nine stars.[40]

### Statistical Analysis

We calculated the OR with 95% CIs in a random-effects model [41] using the metan command in the software Stata 11.0 (Stata Corp, College Station, Tex). The following endpoints were evaluated in our meta-analysis: (1) the pooled OR of colorectal polyps in patients with gastric polyps, (2) the pooled OR of colorectal polyps and neoplasms in patients with gastric neoplasms, and (3) the pooled OR of colorectal neoplasms in patients with duodenal neoplasms. Neoplasms were defined as including benign (adenoma), potentially malignant (pre-cancer), or malignant (cancer). We used the Cochrane Q statistic (*P*<.05 was considered to represent statistically significant heterogeneity) and the *I* ^2^ statistic to assess heterogeneity of ORs among studies. We considered significant heterogeneity exist when *I* ^2^ values were greater than 50%.[42] Publication bias was assessed using Egger's regression test[43] and visual inspection of a funnel plot. Statistical tests were 2 sided and used a significance level of *P*<.05.

## Results

### Literature Search

**Figure 1:**
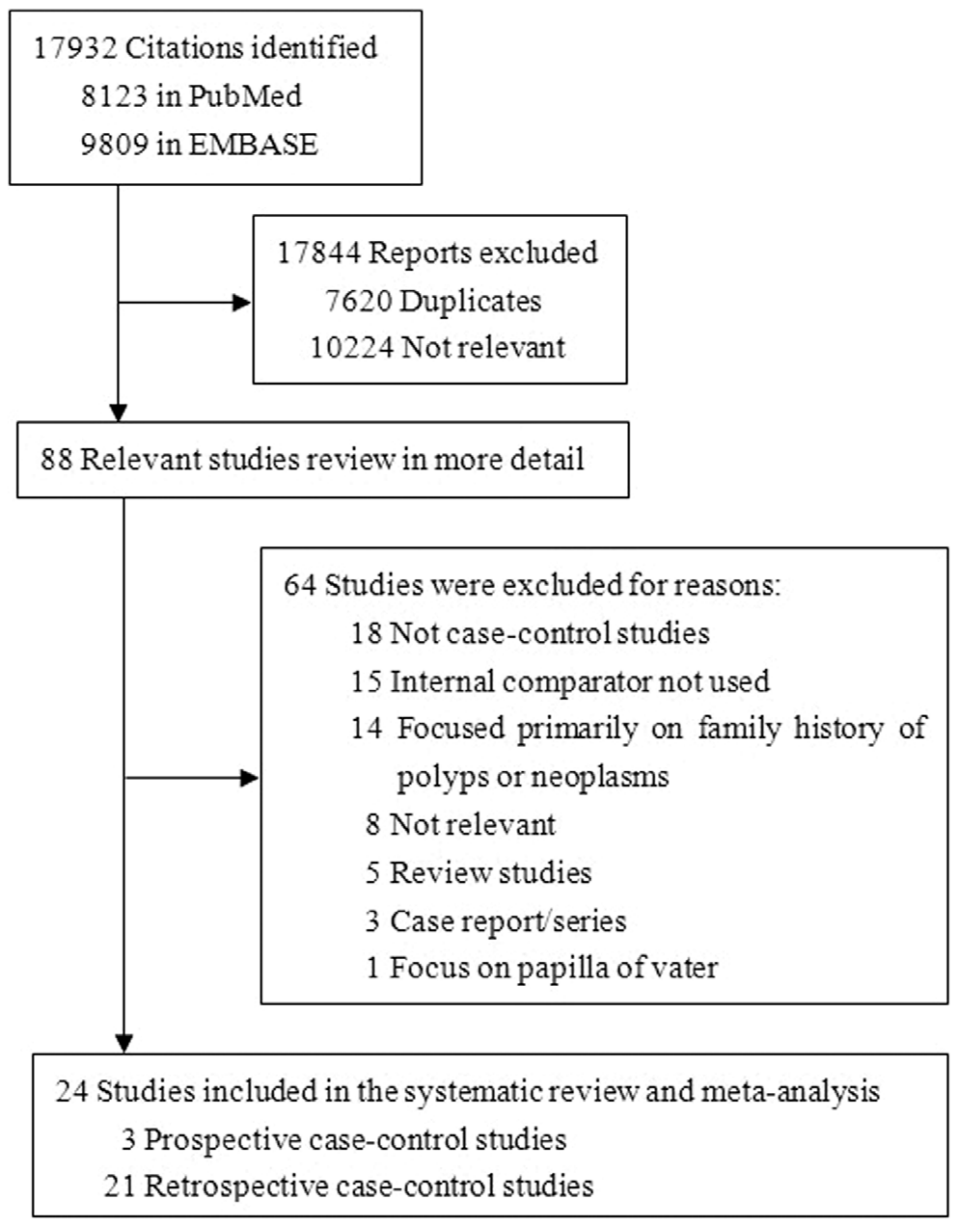
Study Selection Flow Chart.

The search for PubMed and Embase identified a total of 17932 citations. After screening the titles and abstracts with our selection criteria, 7620 were duplicates and 10224 articles were excluded because they did not assess upper GI or colorectal polyps or neoplasms. After reviewing the remaining articles in more detail, 64 articles were excluded for the following reasons. Eighteen studies were excluded because they were not case-control trials and fifteen were excluded because they did not use internal comparator. Fourteen studies were excluded because they focused primarily on family history of polyps or neoplasms; eight studies were not relevant. Five review studies and three case report/series studies were excluded. Specially, one study was excluded because it focuses on neoplasms on papilla of vater[44]. Finally, we included 3 prospective[17], [24], [29] and 21 retrospective[15], [16], [18]–[23], [25]–[28], [30]–[38] case-control studies satisfied the primary selection criteria for this meta-analysis (Figure 1).

### Study Characteristics

Characteristics of the 24 selected studies are shown in Table 1. The studies were conducted in Korea (n = 13 studies), Germany (n = 3), the USA (n = 2), France (n = 2), Australia (n = 1), Netherlands (n = 1), Puerto Rico (n = 1), and Argentina (n = 1). All studies were published between 2000 and 2013 except one conducted in 1995.[16] A total of 37152 participants were assigned in the 24 studies with 5366 cases (with upper GI polyps or neoplasms) and 31786 controls. Sample sizes ranged from 87 to 25687, and the mean age was≥55 years in most studies (n = 18). All studies were case-control design (prospective  = 3, retrospective  = 21). Most studies comprised both men and women except one study[36] including only male patients. Seven studies reported adjusted ORs, twelve reported non-adjusted ORs, and four reported the crude data without adjustment. The results were adjusted for age (6 studies), sex (4 studies), body mass index (BMI) (4 studies), smoking (3 studies), diabetes mellitus (DM) (3 studies), alcohol (2 studies), and use of aspirin or non-steroid anti-inflammatory drugs (2 studies). The study quality scores ranged from 6 to 9 and most studies' score was ≥8 (n = 21)(Table S1).

**Table 1:** Characteristics of the Case-control Studies Included in Meta-analysis.

Authors	Country	Period	Cases' characteristics			Controls' characteristics				Outcome (Types of colorectal diseases)	Adjustment
			No./Male	Cases' type	Mean age, y	No./Male	Controls' type	Mean age, y	Case-control Matching		
Cimmino D.G. et al 2013 [15]	Argentina	2007–2008	78/23	GP	62	169/52	Patients without GP	61	NA	CP, CA, advanced CN^a^	NA
Cappell M. S. et al 1995 [16]	USA	1986–1993	41/NA	GP	67.6	109/NA	Patients without GP	64.6	Age, others^c^	CP, CN	NA
Jung A. et al 2002 [17]	Germany	1998–1999	64/19	Gastric FGP	58	64/24	Patients without FGP	55	Age, sex	CRN	NA
Hwang S.M. et al 2011 [18]	Korea	1992–2007	158/29	Gastric FGP	48.5	2356/1397	Healthy subjects	47.8	NA	CRN	Age, sex
Teichmann J. et al 2008 [19]	Germany	2000–2006	250/NA	Gastric FGP	NA	250/NA	Patients without FGP	NA	Age, sex	CP, CRA, CRC	NA
Genta R.M. et al 2009 [20]	USA	2007–2008	1603/577	Gastric FGP	59	24084/10663	Patients without FGP	58	NA	CRP, CRA, CRC	NA
Bae R.C. et al 2009 [21]	Korea	2005–2008	133/97	GA	61.2	213/162	Health subjects	49.6	NA	CRA, advance CRA^a^	Adjusted OR^b^
Yang M. H. et al 2010 [22]	Korea	2001–2008	87/72	GA	57.2	174/NA	Patients without GA	NA	Age, sex	CRA	NA
Park S. Y. et al 2009 [23]	Korea	2002–2008	221/164	GA	64.4	387/201	Patients without GA	58.3	NA	CRN	Adjusted OR^b^
Park D. I. et al 2010 [24]	Korea	2004–2006	543/362	GC	59.2	1086/724	Patients without GA	58.3	Age, sex, others^d^	CRA, CRC	Age, others^e^
Oh S. Y. et al 2006 [25]	Korea	2002–2004	105/80	GC	59.3	269/189	Patients without GN	57.5	Age, sex	CN	Age, sex, BMI
Lee S. S. et al 2011 [26]	Korea	2005–2010	123/86	GC	62.1	246/172	Patients without GN	62.1	Age, sex	CRN	Age, others^f^
Yoo H. M. et al 2013 [27]	Korea	2009–2010	495/319	GC	60.3	495/319	Healthy subjects	60.3	Age, sex	CRN	NA
Joo M.K. et al 2010 [28]	Korea	2002–2008	186/145	GN	63	186/145	Healthy subjects	63	Age, sex	CRP, advanced CRN^a^	NA
Lee K.J. et al 2011 [29]	Korea	2008–2010	107/78	GN	61.7	107/78	Healthy subjects	61.6	Age, sex	CRN	NA
Park W. et al 2012 [30]	Korea	2005–2010	492/368	GN	61.3	492/286	Healthy subjects	61.3	Age	CRA, CRC, CRN	Age, sex
Kim S. Y. et al 2013 [31]	Korea	2005–2008	416/295	GN	54.1	416/295	Healthy subjects	54.9	Age, sex	CRN	Age, sex, others^g^
Pequin P. et al 2007 [32]	France	1997–2006	35/22	SDN	64.4	70/44	Patients without SDA	63.6	Age, sex	CRN	NA
Lagarde S. et al 2009 [33]	France	1997–2007	29/22	SDA	63.2	58/44	Patients without SDA	62.5	Age, sex	CRN	NA
Ramsoekh D. et al 2008 [34]	Netherlands	1991–2006	49/27	SDA	62.7	147/81	Patients without SDA	63	Age, sex	CRN	NA
Chung W. C. et al 2011 [35]	Korea	2001–2008	26//12	SDA	58.4	78/36	Healthy subjects	58.4	Age, sex	CRN	NA
Gonzalez-Ortiz D. I. et al 2010 [36]	Puerto Rico	1997–2007	21/21	SDA	67	84/NA	Patients without SDA	NA	NA	CA	NA
Dariusz A. et al 2009 [37]	Germany	1990–2006	48/25	SDA	65.7	144/NA	Patients without SDA	NA	Age, sex	CRN	NA
Murray, M. A. et al 2004 [38]	Australia	1992–2002	56/31	SDA	67	102/NA	Patients without SDA	NA	Age, sex	CRN	NA

Abbreviations: BMI, body mass index; CA, colonic adenoma; CN, colonic neoplasm; CP, colonic polyps; CRA, colorectal adenoma; CRC, colorectal cancer; CRN, colorectal neoplasm; CRP, Colorectal polyp; DM, diabetes mellitus; EA, esophageal adenocarcinoma, EC, esophageal cancer; ESCC, esophageal squamous cell carcinomas; FGP, fundic gland polyps; GA, gastric adenoma; GC, gastric cancer; GN, gastric neoplasm; GP, gastric polyps; NA, not available; NSAID, nonsteroid anti-inflammatory drugs; OR, odd ratios; SDA, sporadic duodenal adenomas; SDN, sporadic duodenal neoplasm.

a defined as colorectal neoplasm with a diameter >1 cm, the presence of three or more neoplasm, adenoma with villous component, adenoma with high-grade dysplasia, or adenocarcinoma confirmed by a gastrointestinal pathologist;

b the study reported only an adjusted OR but could offer no additional information;

c including colonoscopy indications;

d including colonoscopy examination, and endoscopist;

e including BMI, smoking, DM, and use of aspirin or NSAID;

f including BMI, smoking, alcohol, DM, and use of aspirin or NSAID;

g including BMI, alcohol, smoking, DM and cholesterol level.

### Risk of colorectal polyps or neoplasms in patients with upper GI polyps or tumors

#### Stomach

**Figure 2:**
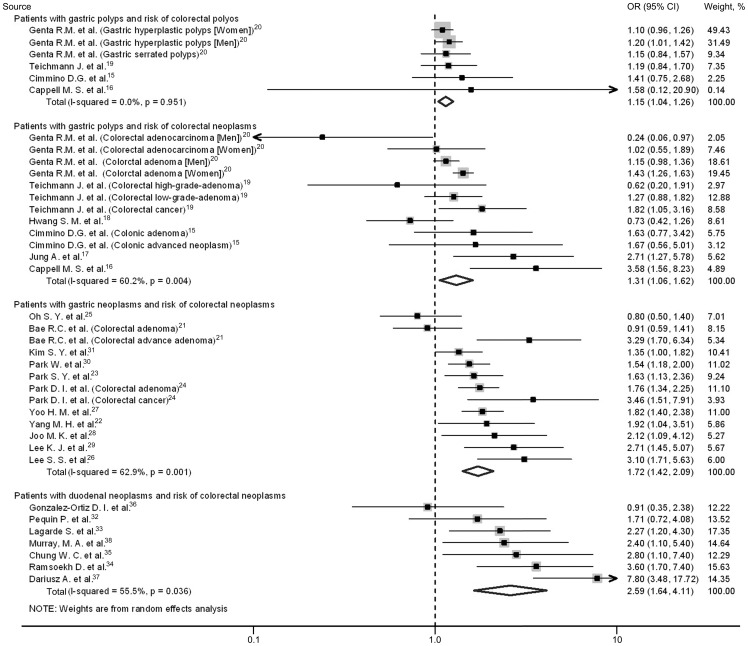
Meta-analysis of risk of colorectal polyps or neoplasms in patients with upper gastrointestinal (stomach and duodenum) polyps or tumors. The varying sizes of the boxes represent the weight in the analysis. Odd ratios (ORs) are derived by a random-effects model using Mantel-Haenszel tests, and error bars indicate 95% confidence intervals (CIs).

Four studies[15], [16], [19], [20] with 1972 cases and 24612 controls compared the risk of colorectal polyps in patients with gastric polyps than those without gastric polyps. The overall prevalence of colorectal polyps was 37.3% (736 of 1972) in cases and 33.9% (8348 of 24612) in controls, yielding a pooled OR of 1.15 (95%CI, 1.04–1.26) (Figure 2). No heterogeneity was found (*I* ^2^ = 0).

Six studies[15]–[20] comprising of 2194 cases and 27032 controls reported the prevalence of colorectal neoplasms in patients with stomach polyps than in those without stomach polyps. The pooled prevalence of colorectal neoplasms was 49.8% (1093 of 2194) in cases and 39.6% (10706 of 27032) in controls, respectively. The estimated summary of OR was 1.31 (95%CI, 1.06–1.62) with significant heterogeneity (*I* ^2^ = 60.2%) (Figure 2). We performed a sensitivity analysis by omitting one study in one time and found that no one study can obviously influence on this result. There was no publication bias detected by Egger's test (*P* = 0.84) and this was also described visually on a symmetrical funnel plot in Figure S1.

Data on the prevalence of colorectal neoplasms in patients with gastric neoplasms comparing with those without stomach neoplasms were available from 11 studies,[21]–[31] which included 5079 cases and 6470 controls. The overall prevalence of colorectal neoplasms was 34.5% (1753 of 5079) in cases and 24.9% (1609 of 6470) in controls, giving an estimated OR of 1.72 (95% CI, 1.42–2.09), with significant heterogeneity (*I* ^2^ = 62.9%) (Figure 2). A sensitivity analysis was conducted by omitting one study in one time and found that no one study can largely impact the result. Both Egger's test (*P* = 0.25) and the symmetrical funnel plot suggested no existence of significant publication bias (Figure S2).

#### Duodenum

There were 7 studies[32]–[38] comprising of 708 cases and 1749 controls revealed risk of colorectal neoplasms in patients with duodenal neoplasm. The pooled prevalence of colorectal neoplasms was 27.7% (196 of 708) in cases and 13.0% (227 of 1749) in controls, respectively. We found a significantly increased risk of colorectal neoplasms in patients with duodenal neoplasm, with pooled OR of 2.59 (95% CI: 1.64–4.11). There was substantial heterogeneity among the studies (*I* ^2^ = 55.5%) (Figure 2). Sensitivity analysis by omitting one study in one time showed that study by Dariusz A. et al[37] obviously affect the result. After dropping this study, the OR become 2.21 (1.56–3.13) with smaller heterogeneity (*I* ^2^ = 10.8%). Visual inspection of the funnel plot showed symmetry, and the Egger's test was not significant (*P* = 0.60) (Figure S3).

### Risk of colorectal neoplasms in patients with gastric cancer with age <50

Two studies reported the prevalence of colorectal neoplasms in patients with gastric cancer with age less than 50. Lee S. S. et al's study[26] showed the prevalence of colorectal neoplasms were 35.8% (6/25) and 17.9% (8/50) in the stomach cancer and the control groups, respectively, yielding an OR of 1.77 (0.42–7.56). Another study by Park D. I. et al[24] reported the gastric cancer group had a significant higher prevalence of colorectal adenoma [32/119 (26.9%) vs. 29/242 (12.0%)], giving an OR of 3.09 (1.61–5.92); and also provide raw data that the prevalence of colorectal cancer were 4 of 119 and zero of 242 in the stomach cancer and the control groups, respectively.

## Discussion

As far as know, this is the first meta-analysis to evaluate the synchronous or heterochronous of colorectal polyps or neoplasms in patients with polyps or tumors in upper GI tract. The principal findings of present review included: (1) OR for colorectal polyps was 1.15 (95% CI, 1.04–1.26) in the gastric polyps group comparing with control groups; (2) Patients with gastric polyps and neoplasms have higher risk (OR, 1.31 [95% CI, 1.06–1.62], and 1.72 [95% CI, 1.42–2.09], respectively) of colorectal neoplasms comparing with their controls; and (3) Positive association was found between the presence of colorectal neoplasms and sporadic duodenal neoplasms (OR, 2.59; 95% CI, 1.64–4.11).

Is there a correlation between upper and lower GI polyps or carcinomas? First, the mechanism underlying this correlation is unknown. As mentioned above, genetic factors and environmental factors may play a role in the etiology of this correlation. Some have hypothesized that this correlation is caused by Helicobacter pylori infections.[45], [46] However, six[15], [20], [21], [27], [28], [31] of the 24 included studies of the present meta-analysis reported Helicobacter pylori status, and all these six studies showed that the present infection of Helicobacter pylori were not associated with colorectal adenoma or cancer. Second, evidence from epidemiologic studies supported this correlation, such as: 1) The prevalence of gastric and duodenal polyps is higher in several colonic polyposis syndromes,[47], [48] and the risk of colonic cancer may be higher in patients with gastric fundic-gland polyps.[19] 2) Patients with adenomas in one location of the GI tract may have additional adenomas in another location.[22], [23] 3) We know that there was a number of (0.7%–1.5%) gastric cancer patients were found to have synchronous or metachronous colorectal cancers[49], [50], and a portion of (2.0%–9.4%) colorectal cancer patients had synchronous or metachronous gastric cancer [51], [52]. Third, results from the present meta-analysis support that patients with upper GI polyps or carcinomas are at a higher risk for lower GI polyps or carcinomas. Recommendation from the American Society for gastrointestinal endoscopy in 2006[8] suggests that both men and women at average risk for developing colorectal cancer should take a screening colonoscopy and then repeat the procedure every 10 years at age of 50 years. Data from our review support that patients with gastric polyps or neoplasms were at increased risk of colorectal polyps or neoplasms, and we recommend these patients should have a screening colonoscopy to detect synchronous or metachronous colorectal lesions, especially those male patients with old age. Interestingly, our review also included two case-controls relative to patients with gastric neoplasms at age of less than 50 years. Lee S. S. et al's study[26] reported the prevalence of colorectal neoplasms were 35.8% (6/25) and 17.9% (8/50) in the gastric cancer and the control groups, respectively. Another study by Park D. I. et al[24] find that the gastric cancer group had a higher prevalence of colorectal adenoma [32/119 (26.9%) vs. 29/242 (12.0%)], and colorectal cancer [4/119 vs. 0/242]. These evidences tend to support patients younger than 50 years with gastric adenoma or cancer should undergo a screening colonoscopy. However, we need more prospective and lager sample researches to test this result.

The prevalence of duodenal adenoma is rare, and its incidence has been estimated at 0.1% to 0.3% in endoscopy series.[53] Duodenal adenoma is commonly associated with familial adenomatous polyposis (FAP). It is uncertain whether patients with duodenal neoplasms without FAP are associated with increased risk of colorectal neoplasms. Seven relevant case-control studies have been published and were included in our meta-analysis. In these studies, patients with a personal or family history of FAP or hereditary nonpolyposis colorectal cancer syndrome, and neoplasms located in the ampulla were excluded. Our meta-analyses showed a statistically significant positive relationship between duodenal neoplasms and colorectal tumors (OR, 2.59; 95% CI, 1.64–4.11). We suggest that all patients with sporadic duodenal adenomas should have screening colonoscopy for earlier detection of colorectal tumors.

The possible limitations of our review must be taken into consideration. First, as with any meta-analysis, our results are limited by the quality and quantity of available evidence on the prevalence of colorectal polyps or neoplasms in patients with upper GI polyps or tumors. Most studies included in our meta-analysis were retrospective case-control design (n = 21 studies), however, these are the best evidence for this issue at present, which may be the foundation for clinicians and patients making decisions. Second, our meta-analysis is limited by the geographical differences, which may play a vital role on the prevalence of gastric and colorectal tumors in Western and Eastern areas. Our pooled results relative to gastric polyps are based on 5 western studies and one Korea study, and pooled results with regard to gastric neoplasms are based upon 11 Korea reports, and the pooled outcome concerning the duodenal neoplasm were achieved from 6 western studies and 1 Korea study. Therefore the applicability of our results is somewhat less useful clinically. Third, we found a significant heterogeneity among studies in some findings of our review. We cannot ruled out some residual or unmeasured confounding coming from various known risk factors, such as sample sizes, H. pylori infection, adjustment, and withdrawal time of the colonoscopy examinations, though the included studies attempted to control for them. However, our meta-analysis restrict to studies that using an internal control group, which is considered as superior in study design[54] and may increase the trustworthiness of our results. Fourth, we could not calculated ORs for the risk of subgroups of colorectal cancer and benign adenomatous tumors because there was no enough data available in the included studies. Finally, unpublished research and missed reports may be present and may have affected our results. However, we included nonEnglish-language studies and publication bias was almost not present in our review.

In conclusion, findings from present meta-analysis of 24 case-control studies suggest that the prevalence of colorectal polyps was higher in patients with gastric polyps than in those without gastric polyps, and the risk of colorectal neoplasms increases significantly in patients with gastric polyps, neoplasms, and duodenal neoplasms. Therefore, screening colonoscopy should be considered for patients with upper GI polyps and neoplasms. Further prospective studies with larger sample size in various regions are necessary to test and verify these results.

## Supporting Information

Figure S1 Funnel plot of studies assessing risk of colorectal neoplasms in patients with stomach polyps than in those without stomach polyps.
(TIF)

Figure S2 Funnel plot of studies assessing risk of colorectal neoplasms in patients with gastric neoplasms comparing with those without stomach neoplasms.
(TIFF)

Figure S3 Funnel plot of studies assessing risk of colorectal neoplasms in patients with duodenal neoplasm comparing with those without duodenal neoplasms.
(TIFF)

Checklist S1 (DOC)

Table S1 Qualities of included studies according to the Newcastle-Ottawa quality assessment scale.
(XLS)
