# Supplementary figures and images for: Clinical Significance of Colonoscopy in Patients with Upper Gastrointestinal Polyps and Neoplasms: A Meta-Analysis
Source: PLoS One. 2014 Mar 17;9(3):e91810. doi: 10.1371/journal.pone.0091810 (PMC3956699; doi:10.1371/journal.pone.0091810)

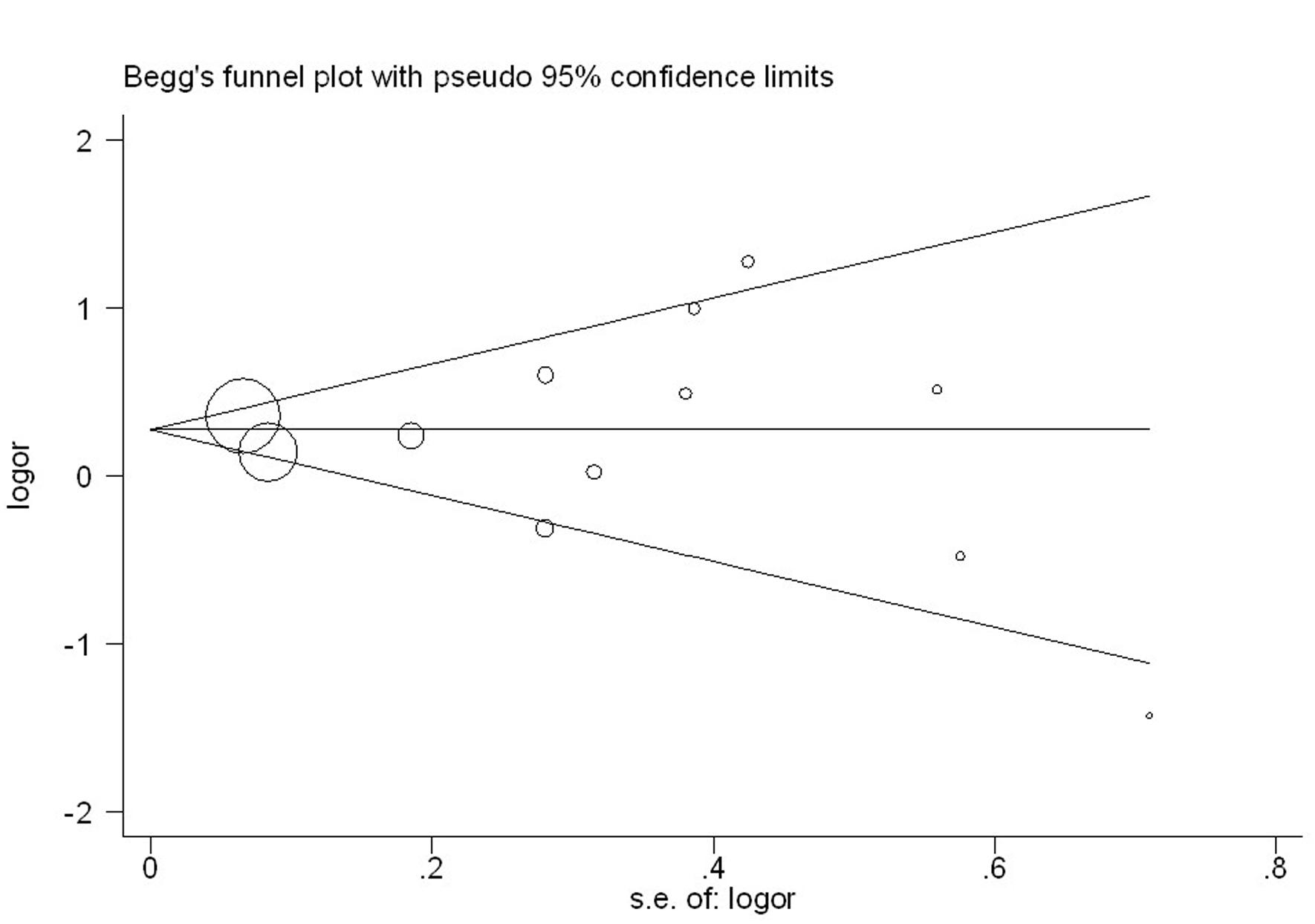

Supplement: Figure S1 — Funnel plot of studies assessing risk of colorectal neoplasms in patients with stomach polyps than in those without stomach polyps. (TIF) [file pone.0091810.s001.tif]

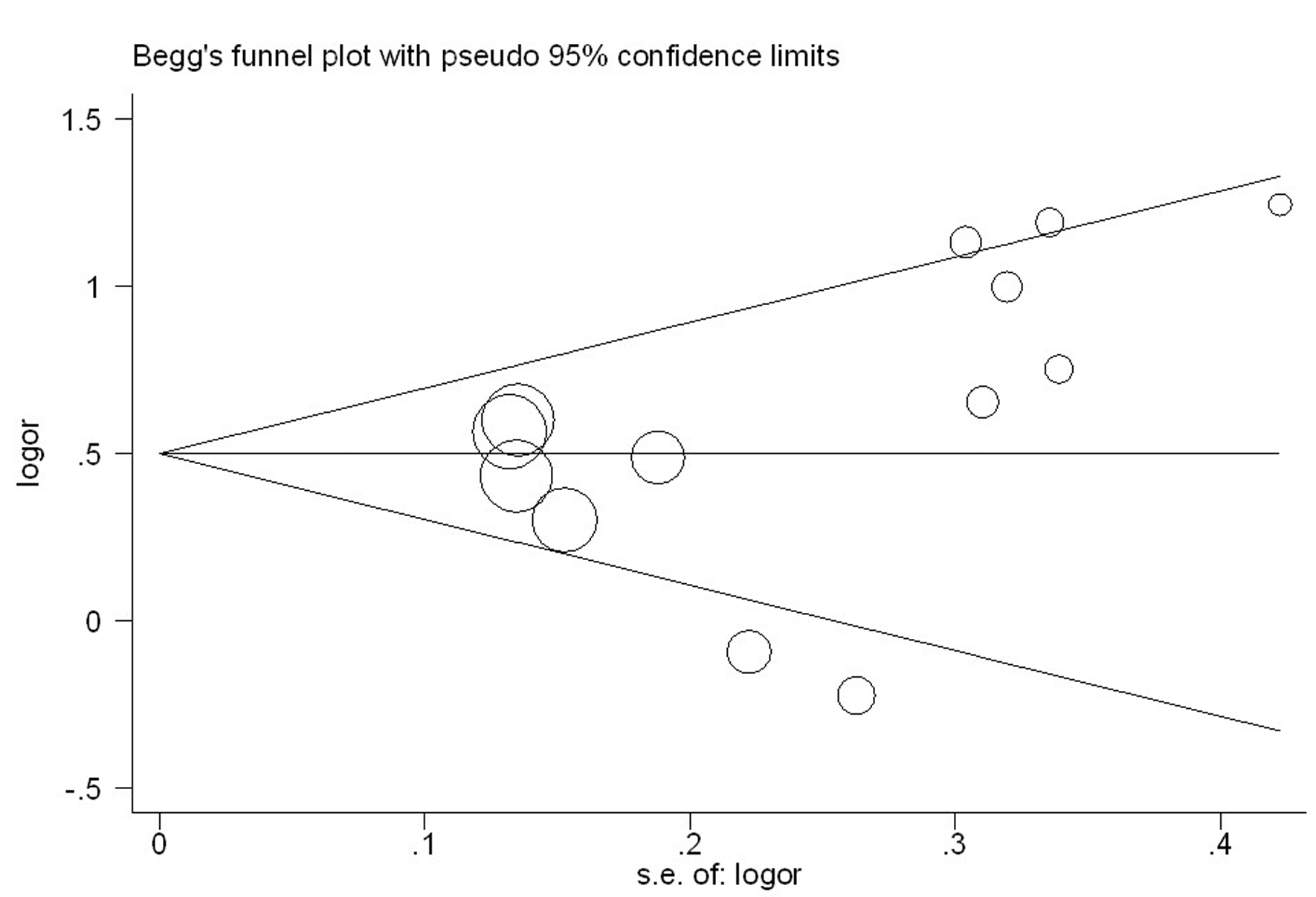

Supplement: Figure S2 — Funnel plot of studies assessing risk of colorectal neoplasms in patients with gastric neoplasms comparing with those without stomach neoplasms. (TIFF) [file pone.0091810.s002.tiff]

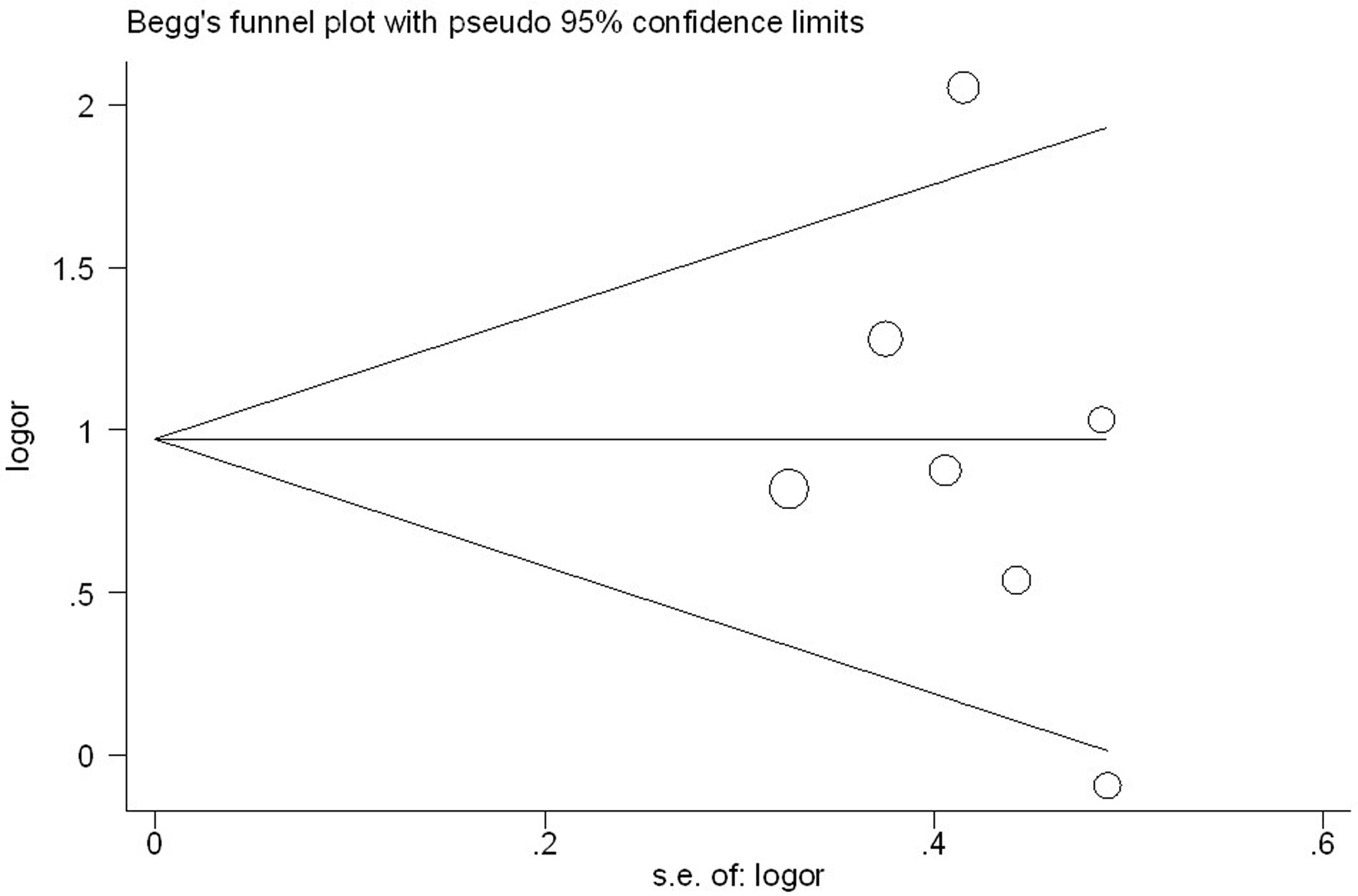

Supplement: Figure S3 — Funnel plot of studies assessing risk of colorectal neoplasms in patients with duodenal neoplasm comparing with those without duodenal neoplasms. (TIFF) [file pone.0091810.s003.tiff]
